# Supplementary material for: Signal sequences target enzymes and structural proteins to bacterial microcompartments and are critical for microcompartment formation
Source: mSphere. 2025 Apr 16;10(5):e00962-24. doi: 10.1128/msphere.00962-24 (PMC12108088; doi:10.1128/msphere.00962-24)
Supplement: Supplemental figures and tables — Tables S1 to S6; Figures S1 to S12. [file msphere.00962-24-s0001.pdf]

Supplementary Information

Signal sequences target enzymes and structural proteins to bacterial microcompartments and are critical for microcompartment formation

Johnson *et al.*

Supplementary Table S1. Strains used in this study.

Strain Number	Organism	Genotype	Abbreviation
DTE003	*S. enterica* serovar Typhimurium LT2	Wild type	WT
CEMS344	*S. enterica* serovar Typhimurium LT2	Δ*pduD*::*pduD*$^{19-*}$	Δ*ssPduD*
CEMS361	*S. enterica* serovar Typhimurium LT2	Δ*pduP*::M-*pduP*$^{18-*}$	Δ*ssPduP*
CEMS362	*S. enterica* serovar Typhimurium LT2	Δ*pduL*::*pduL*$^{17-*}$	Δ*ssPduL*
CEMS351	*S. enterica* serovar Typhimurium LT2	Δ*pduD*::*pduD*$^{19-*}$ Δ*pduP*::*M-pduP*$^{18-*}$	Δ*ssPduDP*
ERJ004	*S. enterica* serovar Typhimurium LT2	Δ*pduD*::*pduD*$^{19-*}$ Δ*pduL*::*pduL*$^{17-*}$	Δ*ssPduDL*
ERJ005	*S. enterica* serovar Typhimurium LT2	Δ*pduP*::M-*pduP*$^{18-*}$ Δ*pduL*::*pduL*$^{17-*}$	Δ*ssPduPL*
CEMS360	*S. enterica* serovar Typhimurium LT2	Δ*pduD*::*pduD*$^{19-*}$ Δ*pduP*::M-*pduP*$^{18-*}$ Δ*pduL*::*pduL*$^{17-*}$	Δ*ssPduDPL*
CMJS256	*S. enterica* serovar Typhimurium LT2	Δ*pocR*	N/A
CEMS179	*S. enterica* serovar Typhimurium LT2	Δ*pduB*	N/A
CEMS342	*S. enterica* serovar Typhimurium LT2	Δ*pduM*	N/A
CEMS375	*S. enterica* serovar Typhimurium LT2	Δ*pduB*::*pduB*$^{\Delta 3-30}$	Δ*ssPduB*
ERJ035	*S. enterica* serovar Typhimurium LT2	Δ*pduM*::M-*pduM*$^{23-*}$	Δ*ssPduM*
ERJ198	*S. enterica* serovar Typhimurium LT2	Δ*pduM*::M-*pduM*$^{23-*}$ Δ*pduB*::*pduB*$^{\Delta 3-30}$	Δ*ssPduMB*
ERJ116	*S. enterica* serovar Typhimurium LT2	Δ*pduD*::*pduD*$^{19-*}$ Δ*pduP*::M-*pduP*$^{18-*}$ Δ*pduL*::*pduL*$^{17-*}$ Δ*pduB*::*pduB*$^{\Delta 3-30}$	Δ*ssPduDPLB*
ERJ039	*S. enterica* serovar Typhimurium LT2	Δ*pduD*::*pduD*$^{19-*}$ Δ*pduP*::M-*pduP*$^{18-*}$ Δ*pduL*::*pduL*$^{17-*}$ Δ*pduM*:: M-*pduM*$^{23-*}$	Δ*ssPduDPLM*
ERJ117	*S. enterica* serovar Typhimurium LT2	Δ*pduD*::*pduD*$^{19-*}$ Δ*pduP*::M-*pduP*$^{18-*}$ Δ*pduL*::*pduL*$^{17-*}$ Δ*pduM*:: M-*pduM*$^{23-*}$ Δ*pduB*::*pduB*$^{\Delta 3-30}$	Δ*ssPduDPLMB*
TMDS107	*S. enterica* serovar Typhimurium LT2	Δ*pduD*::*pduD*$^{1-20}$-*mCherry*	Δ*pduD*::*ssPduD* -*mCherry*
TUC01	*E. coli* W3110	gal490 pglΔ8 λcI857 Δ(cro-bioA) int<>cat/sacB	N/A

Supplementary Table S2. Plasmids used in this study.

Plasmid number	Description	Origin	Resistance
CMJ069	pBAD33t-PduD$^{1-20}$ (ssPduD)-SR-GFPmut2	p15A	Chloramphenicol
EYK208	pBAD33t-PduP$^{1-17}$ (ssPduP)-SR-GFPmut2	p15A	Chloramphenicol
NWKp041	pBAD33t-PduL$^{1-20}$ (ssPduL)-SR-GFPmut2	p15A	Chloramphenicol
EYK193	pBAD-PduG-SR-GFPmut2	p15A	Chloramphenicol
NWKp043	pBAD33t-PduO-GS-GFP	p15A	Chloramphenicol
pBJP017	pBAD33t-PduM$^{1-23}$ (ssPduM)-GFPmut2	p15A	Chloramphenicol
pERJ011	pBAD33t-PduB$^{1-37}$ (ssPduB with linker)-GS-GFPmut2	p15A	Chloramphenicol
NWKp048	pBAD33t-PduA-GS-GFPmut2	p15A	Chloramphenicol
pCEM100	pBAD33t-PduE$^{1-16}$ (ssPduE)-GS-GFPmut2	p15A	Chloramphenicol
pERJ025	GFPmut2	p15A	Chloramphenicol
pERJ012	pBAD33t-PduB$^{1-22}$ (ssPduB without linker)-GS-GFPmut2	p15A	Chloramphenicol
pERJ014	pBAD33t-ssPduE$^{\Delta ESMV::RQII}$-GS-GFPmut2	p15A	Chloramphenicol
pERJ015	pBAD33t-ssPduE$^{\Delta ESMV::QRIV}$-GS-GFPmut2	p15A	Chloramphenicol
pERJ016	pBAD33t-ssPduE$^{\Delta ESMV::ETLI}$-GS-GFPmut2	p15A	Chloramphenicol
pERJ017	pBAD33t-ssPduL$^{\Delta QSTV::ETLI}$-GS-GFPmut2	p15A	Chloramphenicol
pERJ018	pBAD33t-ssPduL$^{\Delta QSTV::RQII}$-GS-GFPmut2	p15A	Chloramphenicol
pERJ019	pBAD33t-ssPduL$^{\Delta QSTV::QRIV}$-GS-GFPmut2	p15A	Chloramphenicol
pCEM119	pBAD33t-PduM-GS-GFPmut2	p15A	Chloramphenicol
pCEM120	pBAD33t-M-PduM^{24-*}-GS-GFPmut2	p15A	Chloramphenicol
pSIM6	λ Red system repressed by cI857	pSC101 *repA*^{ts}	Ampicillin

Supplementary Table S3. Primers used in this study.

Name	Purpose	Description	Sequence (5' → 3')
TMDP021	Sequencing	Amplify from upstream of *pduD* locus (Fwd)	ggcaacaggttatcgcctgc
TMDP022	Sequencing	Amplify from upstream of *pduD* locus (Rev)	ccctgcaggctgttcatgc
TMDP072	Sequencing, recombineering	Amplify from upstream of *pduD* locus (Fwd)	catccagaaagccaagctaacc
TMDP073	Sequencing, recombineering	Amplify from upstream of *pduD* locus (Rev)	cgtccagcgttttattggtgg
TMDP086	Sequencing	Amplify from upstream of *pduP* locus (Fwd)	tcatttacagggaaaagtggtcacc
TMDP087	Sequencing	Amplify from upstream of *pduP* locus (Rev)	tgcgccagaaagccatcg
TMDP088	Sequencing	Amplify from upstream of *pduP* locus (Fwd)	tgtgacctggcggatgc
TMDP089	Sequencing	Amplify from upstream of *pduP* locus (Rev)	tgcagagcctgcatttgc
TMDP043	Sequencing	Amplify from upstream of *pduL* locus (Fwd)	tcagctgcaatctgtgtctgg
TMDP044	Sequencing	Amplify from upstream of *pduL* locus (Rev)	cagaacagtgctggacagtcg
TMDP081	Sequencing	Amplify from upstream of *pduL* locus (Fwd)	ctccgtcattgaacctgagc
TMDP082	Sequencing	Amplify from upstream of *pduL* locus (Rev)	tgagctgtacgcggatgc
oCEM187	Sequencing	Amplify from upstream of *pduM* locus (Fwd)	cgcagcggcatatccatatgtc
oCEM188	Sequencing	Amplify from upstream of *pduM* locus (Rev)	cggtaaacagtgatgtggtgcc
oCEM189	Sequencing	Amplify from upstream of *pduM* locus (Fwd)	gatcgcgggctgattttcaaca
oCEM190	Sequencing	Amplify from upstream of *pduM* locus (Rev)	gatttttgcgtggagacaaccg
oMPV029	Sequencing	Amplify from upstream of *pduB* locus (Fwd)	gtgggtgaagtgaaagccgta
oMPV030	Sequencing	Amplify from upstream of *pduB* locus (Rev)	tccatcgccatcacttcttcg
oERJ006	Sequencing, recombineering	Amplify from upstream of *pduB* locus (Fwd)	GAAAGCCGTACACGTCATCCC
oERJ007	Sequencing, recombineering	Amplify from upstream of *pduB* locus (Rev)	CCTGATTCACAGGGCGTTTCG
oCEM159	Recombineering	Amplify *cat/sacB* with homology upstream of *pduM* (Fwd)	gccgctggtgccgataacccgcatgcctttgcccggctggtaggcccgcgTGTGACGGAAGAT
oCEM160	Recombineering	Amplify *cat/sacB* with homology downstream of *pduM* (Rev)	gatttttgcgtggagacaaccgcgcccgtgactcgtgccagatgcatgatATCAAAGGGAAAA

oCEM171	Recombineering	*pduM* knockout (Fwd)	ctggtgccgataacccgcatgcctttgcccggctggtaggcccgcgatgaaatattcaattaattaagcaggagtaaatcatgcatctggcacgagtcac
oCEM172	Recombineering	*pduM* knockout (Rev)	gtgactcgtgccagatgcatgatttactcctgcttaattaattgaatatttcatcgcgggcct
TMDP003	Recombineering	Amplify *cat/sacB* with homology upstream of *pduP* (Fwd)	CAGCGTTGAGCAGGACATGG
TMDP004	Recombineering	Amplify *cat/sacB* with homology downstream of *pduP* (Rev)	CCAGATGTGCTTATTGGTAAAGCGC
TMDP023	Recombineering	Amplify *cat/sacB* with homology upstream of *pduL* (Fwd)	aaaatgtccgcgccagaagg
TMDP024	Recombineering	Amplify *cat/sacB* with homology downstream of *pduL* (Rev)	cgaagttgcgtaacgctcagc
oCEM161	Recombineering	Amplify $pduD^{19-*}$ with homology upstream of *pduD* (Fwd)	aaacatccctggcgctcttgatcccaacgagattgattaaggggtgagaaatgaagggcagcgataaacc
oCEM162	Recombineering	Amplify $pduD^{19-*}$ with homology downstream of *pduD* (Rev)	gtcccggaccatcgattcaattgcgtcggtattcatggagttatcctttatcaaagcgccacgcg
oCEM163	Recombineering	Amplify $pduP^{18-*}$ with homology upstream of *pduP* (Fwd)	atggacatagcacagaccgccatcgcggctattaacgtgggaactcatcaatgaataccacgccggcg
oCEM164	Recombineering	Amplify $pduP^{18-*}$ with homology downstream of *pduP* (Rev)	accgctgtacaaccgcgtttgtagtgagaaggtattcatcgcgacctcagttagcgaatagaaaagccgttgg
oCEM165	Recombineering	Amplify $pduL^{17-*}$ with homology upstream of *pduL* (Fwd)	ggcgaacctcgtacgctttgcattcattccggcaagcgaggtgaagcgtaatgcgccagcgg
oCEM166	Recombineering	Amplify $pduL^{17-*}$ with homology downstream of *pduL* (Rev)	atgcagccgggagacaatctcctcgacaatgcgctgcagggtttcgccgttcatcgcgggcct
oCEM195	Recombineering	Amplify $pduB^{\Delta 3-30}$ with homology upstream of *pduB* (Fwd)	gccctcacaccgatgtagaaaaaatcttaccgaagggaattagccaatgagccaacctatacgagagacggctatgg
NWKo512	Recombineering	Amplify $pduB^{\Delta 3-30}$ with homology downstream of *pduB* (Rev)	ttcgccagtgcttcaaatcttttcgatctcatgaatcagcctcgtgggtAtcagatgtaggacggacgatcgtttttcg
oERJ001	Golden Gate cloning	Amplify $pduB^{1-37}$ and $pduB^{1-22}$ to add Golden Gate overhang (For)	AGTTACGGTCTCacatgagcagcaatgagctggtgg
oERJ002	Golden Gate cloning	Amplify $pduB^{1-37}$ to add Golden Gate overhang	GTCAACGGTCTCAaaccagccgtctctcgtataggttggg

		compatible with GS linker (Rev)	
oERJ003	Golden Gate cloning	Amplify *pduB*$^{1-22}$ to add Golden Gate overhang compatible with GS linker (Rev)	GTCAACGGTCTCAaaccttccggcgttgccacacg
oBJP094	Golden Gate cloning	Amplify GFPmut2 to add Golden Gate overhang compatible with *pduM*$^{1-23}$ (*ssPduM*) (Fwd)	AttGGTCTCAgagcAgtaaaggagaagaacttttcactgga
oBJP095	Golden Gate cloning	Amplify oBJP097 to add Golden Gate overhang (Fwd)	attaggtctcacatgaacggcgaaaccctgcag
oBJP096	Golden Gate cloning	Amplify oBJP097 to add Golden Gate overhang (Rev)	attaggtctcagctctgggcacggcgatg
oBJP097	Golden Gate cloning	Oligo encoding *pduM*$^{1-23}$ (*ssPduM*)	atgaacggcgaaaccctgcagcgcattgtcgaggagattgtctcccggctgcatcgccgtgcccagagc
oBJP137	Golden Gate cloning	Amplify GFPmut2 to add Golden Gate overhang and GS linker (Fwd)	AttaGGTCTCAggttctAgtaaaggagaagaacttttcactgg
NWKo569	Golden Gate cloning	Amplify *pduA* to add Golden Gate overhang (Fwd)	AttGGTCTCACATGCAACAAGAAGCACTAGG
NWKo570	Golden Gate cloning	Amplify *pduA* to add Golden Gate overhang and GS linker (Rev)	ATTGGTCTCAGCTGCCttggctaattcccttcgg
NWKo506	Golden Gate cloning	Amplify GFPmut2 to add Golden Gate overhang compatible with GS linker (Fwd)	AttGGTCTCACAGCAgtaaaggagaagaacttttcactgg
NWKo507	Golden Gate cloning	Amplify GFPmut2 to add Golden Gate overhang (Rev)	ATTGGTCTCATTTAtttgtatagttcatccatgccatgtg
oCEM207	Golden Gate cloning	Oligo containing *pduE*$^{1-16}$ (*ssPduE*), GS linker, and Golden Gate overhangs	tGGTCTCACatgaataccgacgcaattgaatcgatggtccgggacgtattgagccgcGGCAGCTGAGACCa
oCEM208	Golden Gate cloning	Amplify oCEM207 and oCEM266 - 268 to add buffer regions outside of BsaI cut sites (Fwd)	gggtGGTCTCACatga
oCEM209	Golden Gate cloning	Amplify oCEM207 and oCEM266 - 271 to add buffer regions outside of BsaI cut sites (Rev)	gggtGGTCTCAGCTGC
oCEM266	Golden Gate cloning	Oligo containing *ssPduE*$^{\Delta ESMV::ETLI}$, GS linker, and Golden Gate overhangs	tGGTCTCACatgaataccgacgcaattGAAACTCTTATTcgggacgtattgagccgcGGCAGCTGAGACCa
oCEM267	Golden Gate cloning	Oligo containing *ssPduE*$^{\Delta ESMV::QRIV}$, GS linker, and Golden Gate overhangs	tGGTCTCACatgaataccgacgcaattCAGCGTATTGTAcg

			ggacgtattgagccgcGGCAGCTGAGACCa
oCEM268	Golden Gate cloning	Oligo containing *ssPduE*$^{\Delta ESMV::RQII}$, GS linker, and Golden Gate overhangs	tGGTCTCACatgaataccgacgcaattCGTCAGATTATCcgggacgtattgagccgcGGCAGCTGAGACCa
oCEM265	Golden Gate cloning	Amplify oCEM269 - 271 to add buffer regions outside of BsaI cut sites (Fwd)	gggtGGTCTCACatgg
oCEM269	Golden Gate cloning	Oligo containing *ssPduL*$^{\Delta QSTV::ETLI}$, GS linker, and Golden Gate overhangs	tGGTCTCACatggataaagagcttctgGAAACTCTTATTcgtaaagttctcgacgagGGCAGCTGAGACCa
oCEM270	Golden Gate cloning	Oligo containing *ssPduL*$^{\Delta QSTV::QRIV}$, GS linker, and Golden Gate overhangs	tGGTCTCACatggataaagagcttctgCAGCGTATTGTAcgtaaagttctcgacgagGGCAGCTGAGACCa
oCEM271	Golden Gate cloning	Oligo containing *ssPduL*$^{\Delta QSTV::RQII}$, GS linker, and Golden Gate overhangs	tGGTCTCACatggataaagagcttctgCGTCAGATTATCcgtaaagttctcgacgagGGCAGCTGAGACCa
oCEM240	Golden Gate cloning	Amplify *pduM* to add Golden Gate overhang (Fwd)	AttGGTCTCACATGAACGGCGAAACCCTG
oCEM241	Golden Gate cloning	Amplify *pduM* and *pduM*$^{24-*}$ to add Golden Gate overhang and GS linker (Rev)	ATTGGTCTCAGCTGCCctcctgcttaattaattgaatattccgcg
oCEM242	Golden Gate cloning	Amplify *pduM*$^{24-*}$ to add Golden Gate overhang (Fwd)	AttGGTCTCACATGACGGCGACGCTGAG

Supplementary Table S4. Number of cells counted per replicate for the puncta counts shown in Figures 3b, 4b, and 5b.

		Cells Counted per Replicate			
**Genotype**	**Reporter**	**1**	**2**	**3**	**Total**
WT	ssPduD-GFP	78	69	84	231
WT	ssPduP-GFP	113	79	66	258
WT	ssPduL-GFP	72	142	58	272
WT	PduG-GFP	93	166	106	365
WT	PduO-GFP	160	169	116	445
WT	ssPduM-GFP	211	68	90	369
WT	PduA-GFP	72	66	59	197
Δ*ssPduD*	ssPduD-GFP	69	92	112	273
Δ*ssPduD*	ssPduP-GFP	86	90	103	279
Δ*ssPduD*	ssPduL-GFP	82	70	139	291
Δ*ssPduD*	PduG-GFP	85	71	47	203
Δ*ssPduD*	PduO-GFP	115	118	124	357
Δ*ssPduD*	PduA-GFP	96	95	73	264

Δ*ssPduP*	ssPduD-GFP	45	99	88	232
Δ*ssPduP*	ssPduP-GFP	88	88	74	250
Δ*ssPduP*	ssPduL-GFP	82	130	147	359
Δ*ssPduP*	PduG-GFP	110	118	92	320
Δ*ssPduP*	PduO-GFP	142	65	91	298
Δ*ssPduP*	PduA-GFP	139	44	92	275
Δ*ssPduL*	ssPduD-GFP	77	84	146	307
Δ*ssPduL*	ssPduP-GFP	72	72	126	270
Δ*ssPduL*	ssPduL-GFP	94	82	76	252
Δ*ssPduL*	PduG-GFP	57	59	84	200
Δ*ssPduL*	PduO-GFP	112	73	88	273
Δ*ssPduL*	PduA-GFP	143	155	122	420
Δ*ssPduDP*	ssPduD-GFP	77	105	66	248
Δ*ssPduDP*	ssPduP-GFP	71	117	117	305
Δ*ssPduDP*	ssPduL-GFP	100	120	157	377
Δ*ssPduDP*	PduG-GFP	67	56	146	269
Δ*ssPduDP*	PduO-GFP	70	131	124	325
Δ*ssPduDP*	PduA-GFP	163	66	59	288
Δ*ssPduDL*	ssPduD-GFP	132	69	64	265
Δ*ssPduDL*	ssPduP-GFP	83	74	148	305
Δ*ssPduDL*	ssPduL-GFP	88	101	86	275
Δ*ssPduDL*	PduG-GFP	91	173	83	347
Δ*ssPduDL*	PduO-GFP	81	110	115	306
Δ*ssPduDL*	PduA-GFP	188	94	72	354
Δ*ssPduPL*	ssPduD-GFP	74	97	114	285
Δ*ssPduPL*	ssPduP-GFP	92	93	138	323
Δ*ssPduPL*	ssPduL-GFP	105	62	76	243
Δ*ssPduPL*	PduG-GFP	75	136	99	310
Δ*ssPduPL*	PduO-GFP	70	65	96	231
Δ*ssPduPL*	PduA-GFP	130	127	113	370
Δ*ssPduDPL*	ssPduD-GFP	115	89	113	317
Δ*ssPduDPL*	ssPduP-GFP	79	73	119	271
Δ*ssPduDPL*	ssPduL-GFP	100	93	57	250
Δ*ssPduDPL*	PduG-GFP	95	54	98	247
Δ*ssPduDPL*	PduO-GFP	99	81	134	314
Δ*ssPduDPL*	PduA-GFP	104	108	61	273
Δ*pduB*	ssPduD-GFP	135	214	275	624
Δ*pduB*	ssPduM-GFP	43	104	111	258
Δ*pduB*	PduG-GFP	232	311	233	776
Δ*pduB*	PduA-GFP	40	33	179	252
Δ*ssPduB*	ssPduD-GFP	110	60	172	342
Δ*ssPduB*	ssPduM-GFP	268	108	108	484
Δ*ssPduB*	PduG-GFP	166	53	57	276
Δ*ssPduB*	PduA-GFP	82	124	137	343
Δ*pduM*	ssPduD-GFP	231	205	216	652

Δ*pduM*	ssPduM-GFP	129	168	221	518
Δ*pduM*	PduG-GFP	159	167	157	483
Δ*pduM*	PduA-GFP	149	111	60	320
Δ*ssPduM*	ssPduD-GFP	96	42	108	246
Δ*ssPduM*	ssPduM-GFP	80	92	178	350
Δ*ssPduM*	PduG-GFP	238	119	136	493
Δ*ssPduM*	PduA-GFP	33	50	58	141
Δ*ssPduMB*	ssPduD-GFP	55	76	100	231
Δ*ssPduMB*	ssPduM-GFP	152	52	109	313
Δ*ssPduMB*	PduG-GFP	81	159	55	295
Δ*ssPduMB*	PduA-GFP	68	46	106	220
Δ*ssPduDPLB*	ssPduD-GFP	79	72	127	278
Δ*ssPduDPLB*	ssPduM-GFP	37	113	47	197
Δ*ssPduDPLB*	PduG-GFP	80	84	93	257
Δ*ssPduDPLB*	PduA-GFP	59	46	54	159
Δ*ssPduDPLM*	ssPduD-GFP	103	109	82	294
Δ*ssPduDPLM*	ssPduM-GFP	90	45	91	226
Δ*ssPduDPLM*	PduG-GFP	230	93	161	484
Δ*ssPduDPLM*	PduA-GFP	108	97	88	293
Δ*ssPduDPLMB*	ssPduD-GFP	58	131	132	321
Δ*ssPduDPLMB*	ssPduM-GFP	73	117	88	278
Δ*ssPduDPLMB*	PduG-GFP	105	140	163	408
Δ*ssPduDPLMB*	PduA-GFP	86	178	208	472

Supplementary Table S5. Designs and results of statistical tests conducted in this study.

Test description	One-factor ANOVA between puncta counts of core reporters expressed in a strain, Bonferroni post-hoc test
Reporters included in test	ssPduD-GFP, ssPduP-GFP, ssPduL-GFP, PduG-GFP, PduO-GFP
F statistic and *p* value, Δ*ssPduD*	F = 129.08, p = 1.462×10^{-8}
F statistic and *p* value, Δ*ssPduP*	F = 9.41, p = 0.0020
F statistic and *p* value, Δ*ssPduL*	F = 7.67, p = 0.0043
F statistic and *p* value, Δ*ssPduDP*	F = 183.13, p = 2.628×10^{-9}
F statistic and *p* value, Δ*ssPduDL*	F = 104.58, p = 4.080×10^{-8}
F statistic and *p* value, Δ*ssPduPL*	F = 4.18, p = 0.0303

F statistic and *p* value, Δ*ssPduDPL*	$F = 27.41$, $p = 2.278\times10^{-5}$
Total degrees of freedom	14 (Between groups df = 4, within groups df = 10)
Test description	One-factor ANOVA between puncta counts of different reporters expressed in a strain, Dunnett post-hoc test
Control group	PduA-GFP
Experimental groups	ssPduD-GFP, ssPduM-GFP, PduG-GFP
F statistic and *p* value, Δ*pduM*	$F = 21.94$, $p = 3.25\times10^{-4}$
F statistic and *p* value, Δ*ssPduM*	$F = 25.15$, $p = 2.00\times10^{-4}$
F statistic and *p* value, Δ*ssPduDPLB*	$F = 37.37$, $p = 4.71\times10^{-5}$
F statistic and *p* value, Δ*ssPduDPLMB*	$F = 36.12$, $p = 5.35\times10^{-5}$
Total degrees of freedom	11 (Between groups df = 3, within groups df = 8)
Control group	ssPduD-GFP
Experimental groups	ssPduM-GFP, PduG-GFP
F statistic and *p* value, Δ*ssPduDPLB*	$F = 0.38$, $p = 0.70$
F statistic and *p* value, Δ*ssPduDPLM*	$F = 0.76$, $p = 0.51$
F statistic and *p* value, Δ*ssPduDPLMB*	$F = 1.62$, $p = 0.27$
Total degrees of freedom	8 (Between groups df = 2, within groups df = 6)
Test description	One-factor ANOVA between puncta counts of PduA-GFP expressed in different strains, Bonferroni post-hoc test
Strains included in test	WT, Δ*pduB*, Δ*ssPduB*, Δ*pduM*, Δ*ssPduM*, Δ*ssPduMB*, Δ*ssPduDPLB*, Δ*ssPduDPLM*, Δ*ssPduDPLMB*
F statistic and *p* value	$F = 26.32$, $p = 2.06\times10^{-8}$
Total degrees of freedom	26 (Between groups df = 8, within groups df = 18)
Test description	Two-factor ANOVA between puncta counts of reporters expressed in two strains
Strains included in test	Δ*ssPduM*, Δ*pduM*
Reporters included in test	ssPduD-GFP, ssPduM-GFP, PduG-GFP, PduA-GFP
F statistic and *p* value, strains	$F = 38.96$, $p = 1.18\times10^{-5}$
F statistic and *p* value, reporters	$F = 38.24$, $p = 1.59\times10^{-7}$

F statistic and *p* value, interaction	$F = 7.17$, $p = 0.0029$
Total degrees of freedom	23 (Between strains df = 1, between reporters df = 3, interaction df = 3, within groups df = 16)
Simple main effects test description	Two-tailed Student's *t*-test between strains for each reporter
t statistic and *p* value, ssPduD-GFP	$t = 12.31$, $p = 2.50\times10^{-4}$
t statistic and *p* value, ssPduM-GFP	$t = 2.21$, $p = 0.091$
t statistic and *p* value, PduG-GFP	$t = 0.76$, $p = 0.49$
t statistic and *p* value, PduA-GFP	$t = 3.06$, $p = 0.0376$
Degrees of freedom, simple main effects *t*-tests	4

Supplementary Table S6. Bonferroni post hoc test from ANOVA used in Figure 3b. This table shows pairwise *p*-values between normalized puncta counts of enzymatic signal sequences and other core reporters in enzymatic signal sequence knockout strains.

Reporter 1	Reporter 2	*p*, Δ*ssD*	*p*, Δ*ssP*	*p*, Δ*ssL*	*p*, Δ*ssDP*	*p*, Δ*ssDL*	*p*, Δ*ssPL*	*p*, Δ*ssDPL*
ssPduD-GFP	ssPduP-GFP	8.65×10^{-7}	1	1	2.01×10^{-7}	1.33×10^{-7}	1	6.84×10^{-5}
ssPduD-GFP	ssPduL-GFP	8.14×10^{-7}	1	0.3579	8.76×10^{-9}	1.56×10^{-6}	0.130	6.25×10^{-3}
ssPduD-GFP	PduG-GFP	2.32×10^{-8}	1	1	9.16×10^{-9}	6.03×10^{-8}	1	2.85×10^{-5}
ssPduD-GFP	PduO-GFP	2.77×10^{-8}	4.41×10^{-3}	0.1545	6.75×10^{-9}	3.57×10^{-7}	1	4.54×10^{-3}
ssPduP-GFP	ssPduL-GFP	1	1	0.0320	1.28×10^{-3}	0.0379	1	0.0477
ssPduP-GFP	PduG-GFP	1.03×10^{-3}	1	1	1.46×10^{-3}	1	1	1
ssPduP-GFP	PduO-GFP	1.64×10^{-3}	6.93×10^{-3}	0.0146	5.96×10^{-4}	1	0.429	0.0687
ssPduL-GFP	PduG-GFP	1.15×10^{-3}	1	0.0795	1	4.24×10^{-3}	0.6156	0.0118
ssPduL-GFP	PduO-GFP	1.85×10^{-3}	0.0322	1	1	0.582	0.0393	1
PduG-GFP	PduO-GFP	1	5.07×10^{-3}	0.0352	1	0.1284	1	0.0166

a

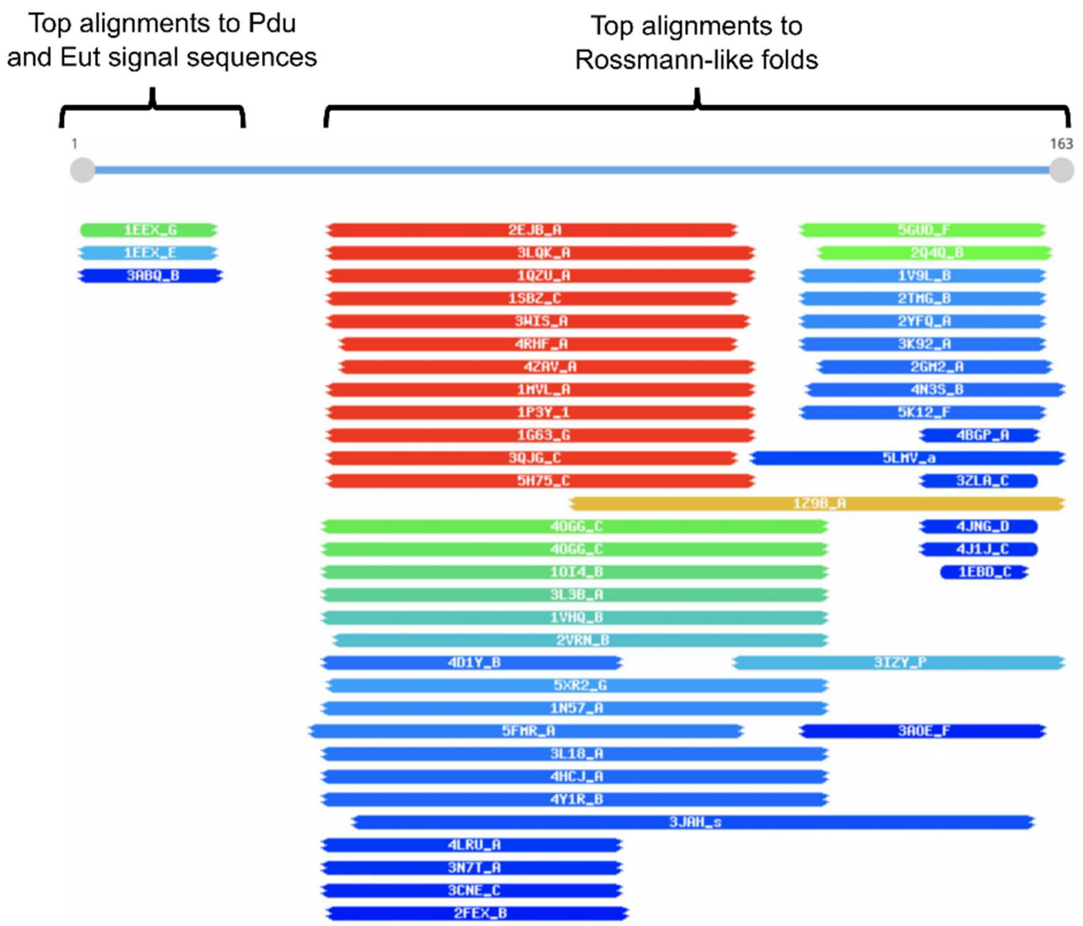

b

Top 3 HHpred hits for the PduM N-terminus

Protein Data Bank ID	Description	Domains
1EEX_G	Propanediol dehydrase small subunit (PduE) from *Klebsiella oxytoca*	Dehydratase small subunit (pfam02287)
1EEX_E	Propanediol dehydrase medium subunit (PduD) from *Klebsiella oxytoca*	Dehydratase medium subunit (pfam02288)
3ABQ_B	Ethanolamine ammonia lyase small subunit (EutC) from *Escherichia coli*	Ethanolamine ammonia lyase light chain (pfam05985)

Top 3 HHpred hits for the body of PduM

Protein Data Bank ID	Description	Domains
2EJB_A	Flavin prenyltransferase (UbiX) from *Aquifex aeolicus*	Flavoprotein (pfam02441)
3LQK_A	Dipicolinate synthase subunit B from *Halalkalibacterium halodurans*	Flavoprotein (pfam02441)
1QZU_A	Phosphopantothenoylcysteine decarboxylase from *Homo sapiens*	Flavoprotein (pfam02441)

Supplementary Figure S1. (a) HHpred results for PduM, indicating that the N-terminus of PduM structurally aligns with MCP signal sequences while the body of PduM structurally aligns with proteins with Rossmann-like folds. Hits shown in red have stronger structural homology, and hits shown in blue have weaker structural homology. This analysis was performed in April 2018 using default HHpred settings (1). (b) Functions, source organisms, and pfam domains of the top 3 HHpred hits for the PduM N-terminus and the body of PduM.

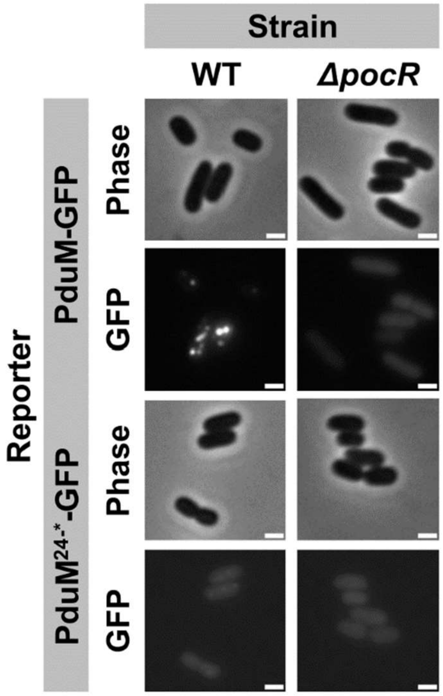

Supplementary Figure S2. Optical and fluorescence micrographs of full length PduM and PduM^{24-*} (PduM without its signal sequence) fused to GFPmut2. These constructs were overexpressed both in wild-type (WT) MCP-forming *S. enterica* and in Δ*pocR*, an assembly-deficient *S. enterica* strain in which all MCP formation is abolished. All scale bars are 1 μm. Similar results were observed across at least three biological replicates of each strain.

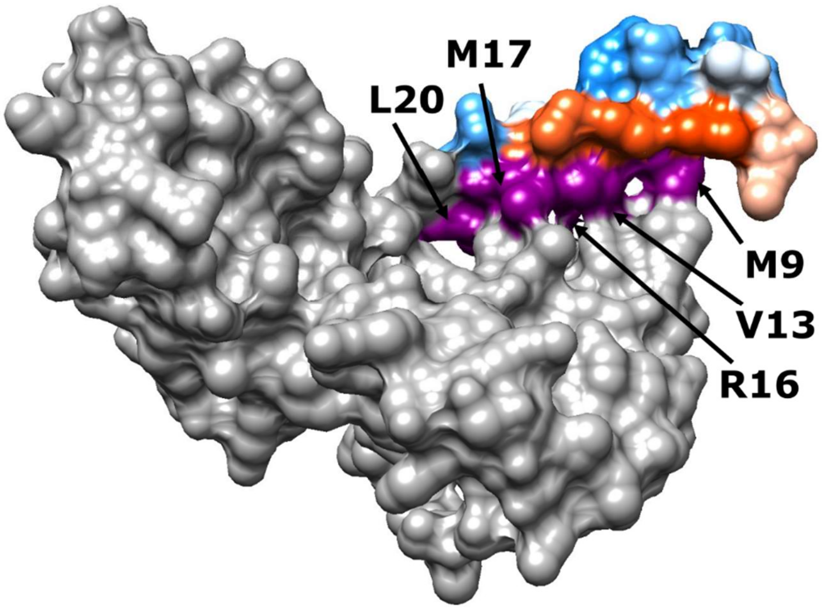

Supplementary Figure S3. Predicted hydrophobicity surface of PduE from *S. enterica* LT2. The body of PduE is colored gray, and the signal sequence-like N-terminal motif (ssPduE) is colored in blue, red, and purple. Hydrophilic areas are shown in blue, hydrophobic areas are shown in red, and residues that are predicted to connect ssPduE to the body of PduE are shown in purple and labeled with arrows. This structure was downloaded from the AlphaFold Protein Structure Database and visualized using UCSF Chimera (2-4).

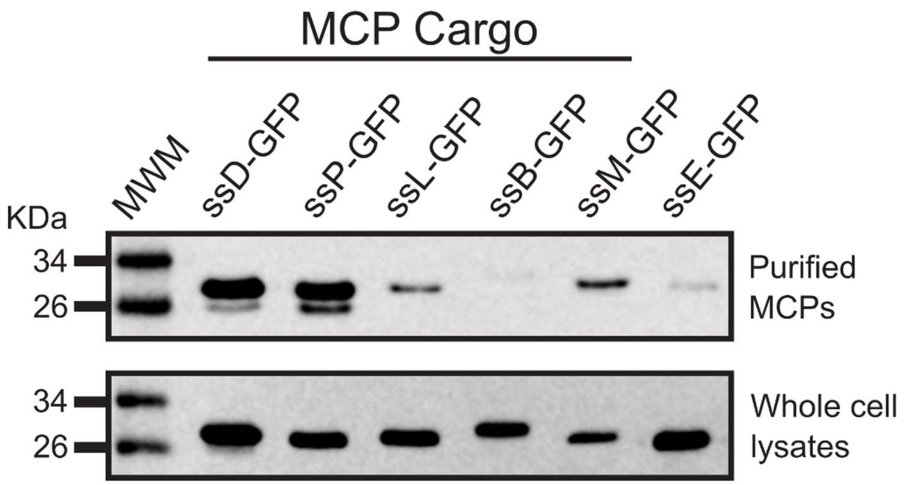

Supplementary Figure S4. Anti-GFP western blots of purified MCPs and whole cell lysates from wild-type *Salmonella enterica* overexpressing each signal sequence fused to GFPmut2. Similar results were observed across two independent biological replicates. The purified Pdu MCPs samples used in this blot had protein concentrations (measured by bicinchoninic acid assay) ranging from 0.55 mg/mL to 2.14 mg/mL before normalization.

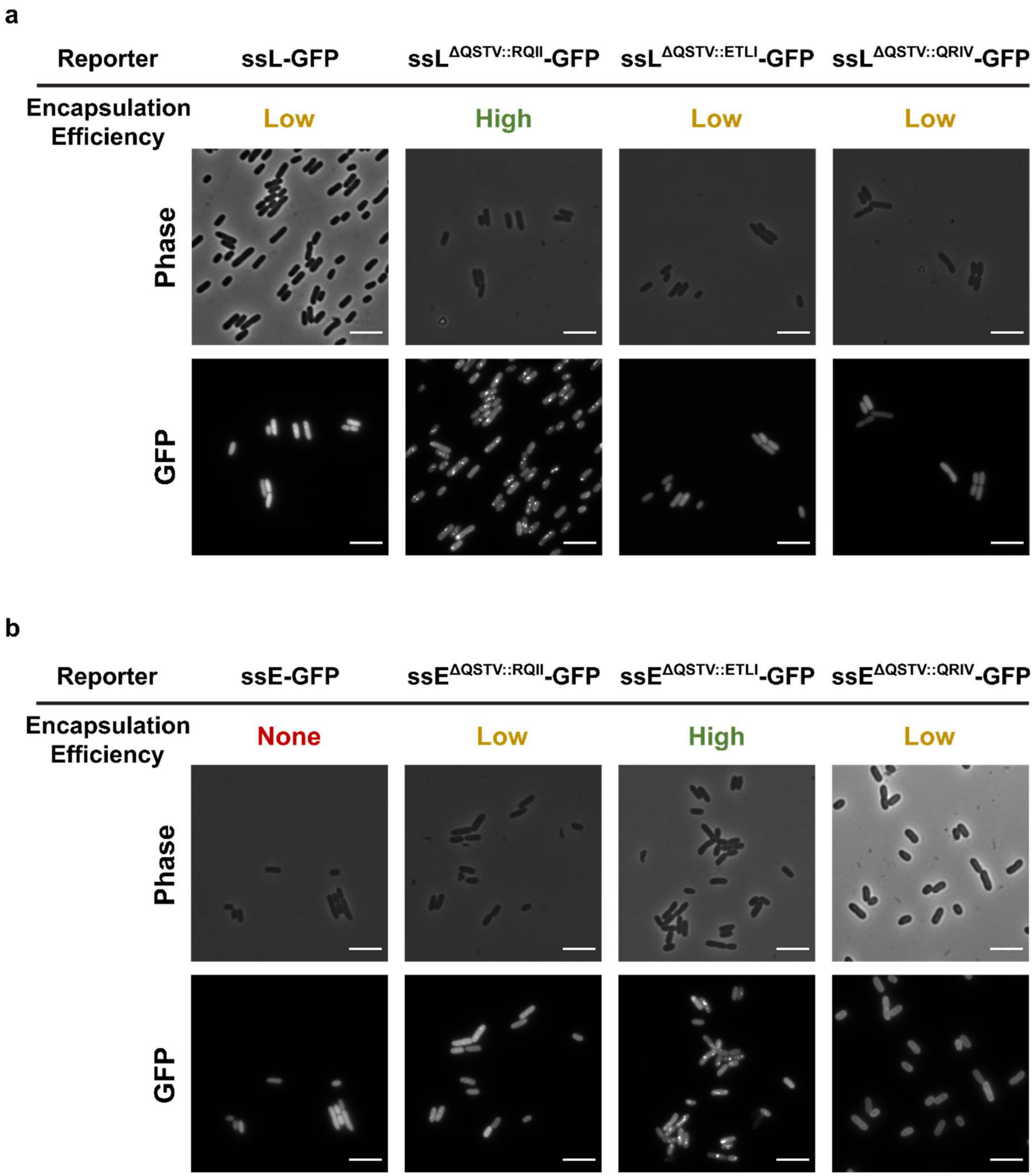

Supplementary Figure S5. Optical and fluorescence micrographs of ssPduL and ssPduE mutants fused to GFPmut2. These constructs were overexpressed in MCP-forming wild type *S. enterica.* These are larger-scale images of the same strains shown in Figure 3b. All scale bars are 5 µm. Similar results were observed across at least three biological replicates.

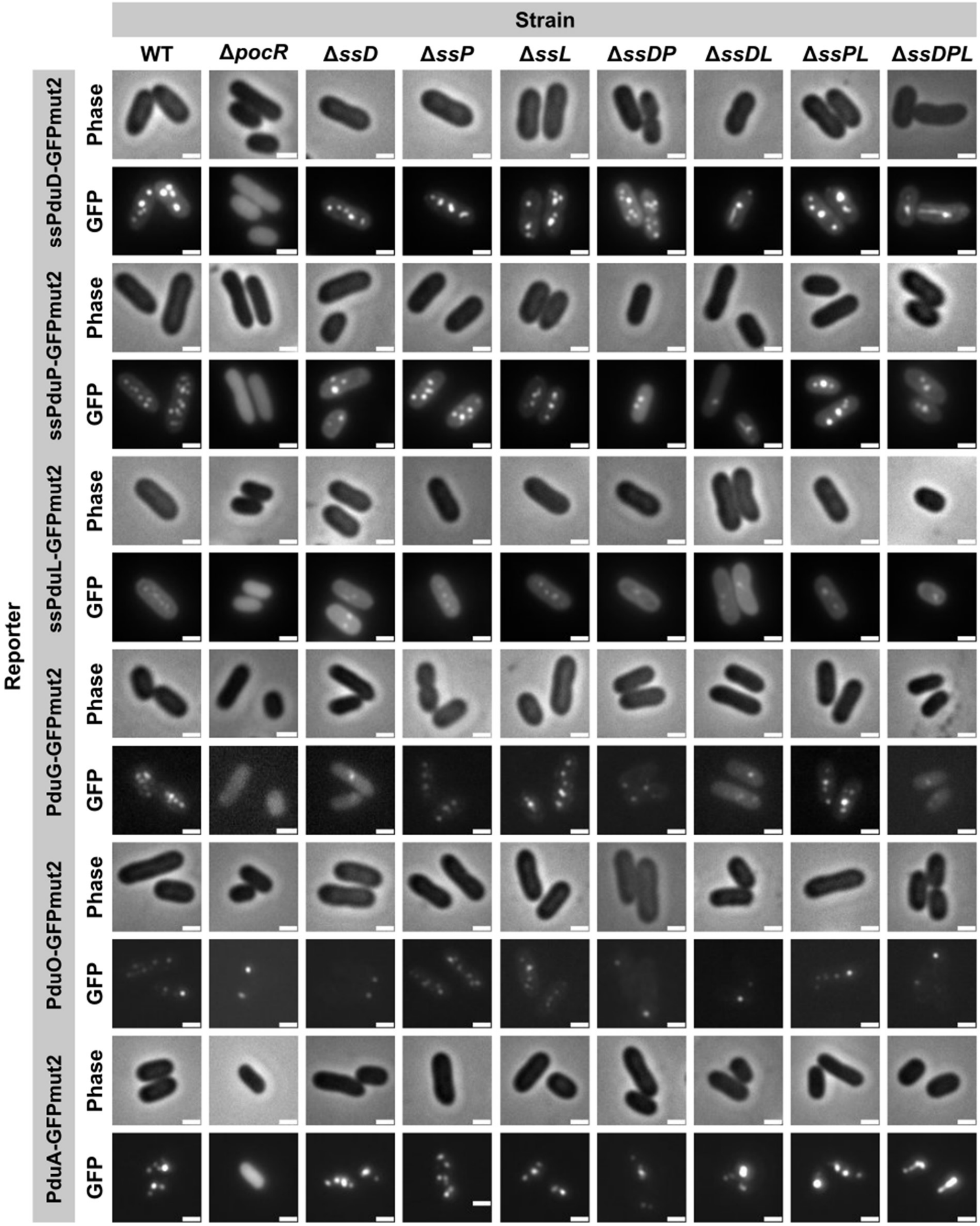

Supplementary Figure S6. Optical and fluorescence micrographs of Pdu MCP core and shell proteins and signal sequences fused to GFPmut2. These constructs were expressed in wild type (WT) *S. enterica*, in Δ*pocR*, an assembly-deficient strain in which all MCP formation is abolished, and in the enzymatic signal sequence knockout strains. All scale bars are 1 μm. Similar results were observed across at least three biological replicates of each strain.

a

	WT	Δ*ssD*	Δ*ssP*	Δ*ssL*	Δ*ssDP*	Δ*ssDL*	Δ*ssPL*	Δ*ssDPL*
ssD-GFP	3.7 ± 0.3	4.0 ± 0.2	3.3 ± 0.2	2.74 ± 0.09	3.97 ± 0.08	2.47 ± 0.09	2.7 ± 0.1	1.75 ± 0.07
ssP-GFP	4.9 ± 0.2	2.8 ± 0.4	4.6 ± 0.01	3.3 ± 0.2	2.6 ± 0.3	1.47 ± 0.09	3.3 ± 0.1	1.3 ± 0.2
ssL-GFP	3.1 ± 0.2	1.72 ± 0.04	3.0 ± 0.3	2.7 ± 0.3	1.0 ± 0.1	1.18 ± 0.06	1.9 ± 0.1	1.10 ± 0.03
PduG-GFP	4.0 ± 0.5	1.3 ± 0.1	3.63 ± 0.06	2.8 ± 0.2	1.3 ± 0.3	1.1 ± 0.2	2.8 ± 0.1	1.00 ± 0.03
PduO-GFP	3.3 ± 0.1	1.09 ± 0.09	3.9 ± 0.3	2.9 ± 0.1	1.00 ± 0.03	1.10 ± 0.05	2.5 ± 0.3	1.2 ± 0.1
PduA-GFP	3.8 ± 0.4	3.1 ± 0.4	4.0 ± 0.6	2.9 ± 0.1	2.7 ± 0.1	1.8 ± 0.1	2.5 ± 0.1	2.1 ± 0.1

b

	WT	Δ*pduB*	Δ*ssB*	Δ*pduM*	Δ*ssM*	Δ*ssMB*	Δ*ssDPLB*	Δ*ssDPLM*	Δ*ssDPLMB*
ssD-GFP	3.7 ± 0.3	0.92 ± 0.04	1.1 ± 0.1	2.4 ± 0.1	1.52 ± 0.03	1.00 ± 0.02	1.05 ± 0.08	1.4 ± 0.2	0.82 ± 0.08
PduG-GFP	4.0 ± 0.5	1.01 ± 0.02	0.99 ± 0.04	1.81 ± 0.06	1.7 ± 0.2	0.97 ± 0.04	1.04 ± 0.04	1.6 ± 0.1	0.90 ± 0.10
ssM-GFP	4.7 ± 0.4	0.94 ± 0.01	0.96 ± 0.02	2.04 ± 0.07	1.9 ± 0.1	0.98 ± 0.03	1.01 ± 0.05	1.5 ± 0.3	0.94 ± 0.05
PduA-GFP	3.8 ± 0.4	3.6 ± 0.3	3.3 ± 0.1	3.3 ± 0.5	2.5 ± 0.2	3.1 ± 0.2	2.0 ± 0.3	1.5 ± 0.1	1.8 ± 0.2

Supplementary Figure S7. Means and standard deviations of GFP puncta per cell for (a) strains and reporters shown in the main figure 3b heatmap and (b) strains and reporters shown in the main figures 4b and 5b heatmaps. The values shown in this figure are the means and standard deviations over three biological replicates of at least 30 cells each and are not normalized to wild type puncta counts.

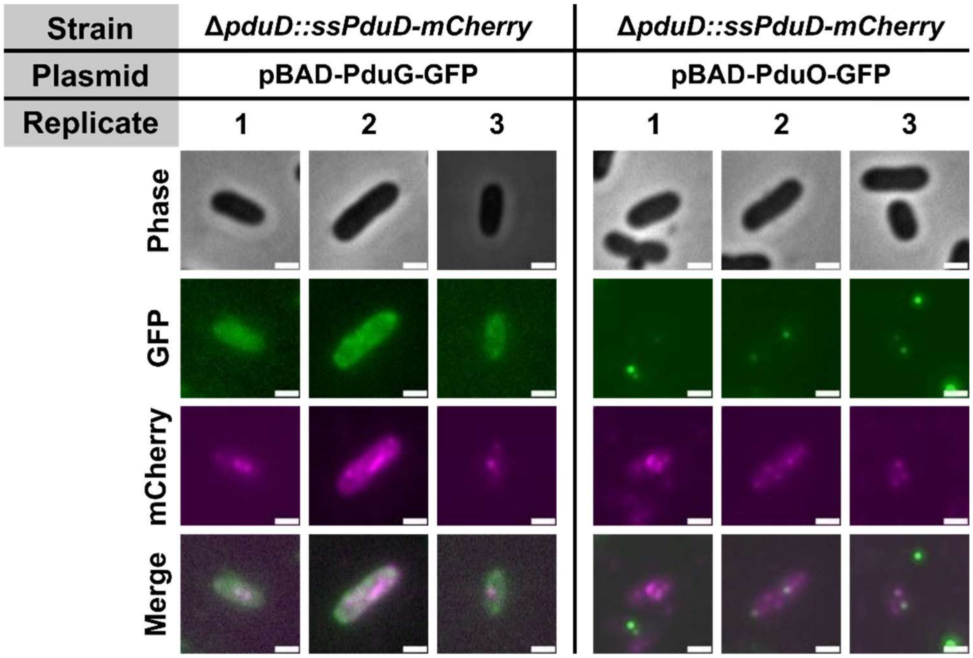

Supplementary Figure S8. Optical and fluorescence micrographs of LT2 strains coexpressing ssPduD-mCherry from the *pduD* locus (Δ*pduD*::*ssPduD-mCherry*) and PduG-GFPmut2 or PduO-GFPmut2 from pBAD33t plasmids. GFP fluorescence is pseudocolored green, and mCherry fluorescence is pseudocolored magenta. The merged images show GFP and mCherry fluorescence overlaid on each other. Representative images are shown from three biological replicates for each strain. While we observed a high degree of cell-to-cell variability in mCherry expression, these images are representative of cells with high mCherry expression. All scale bars are 1 μm.

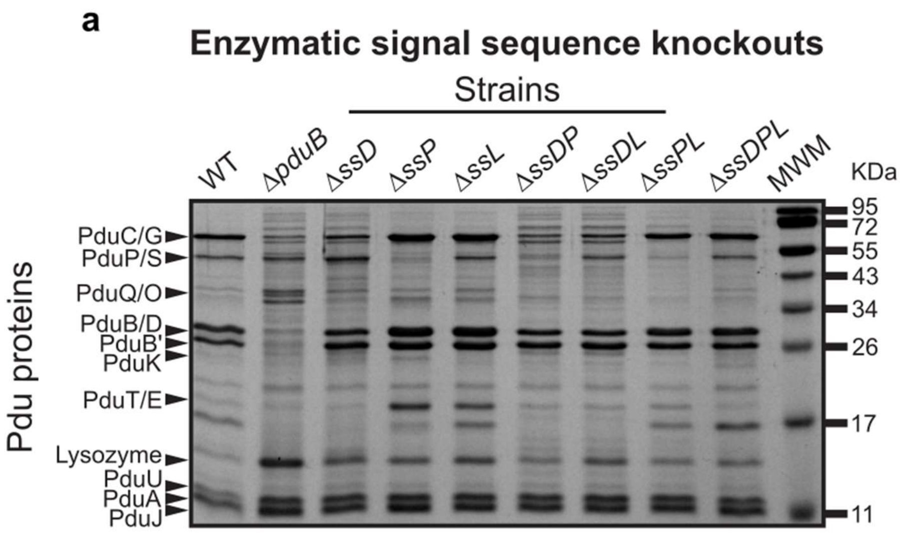

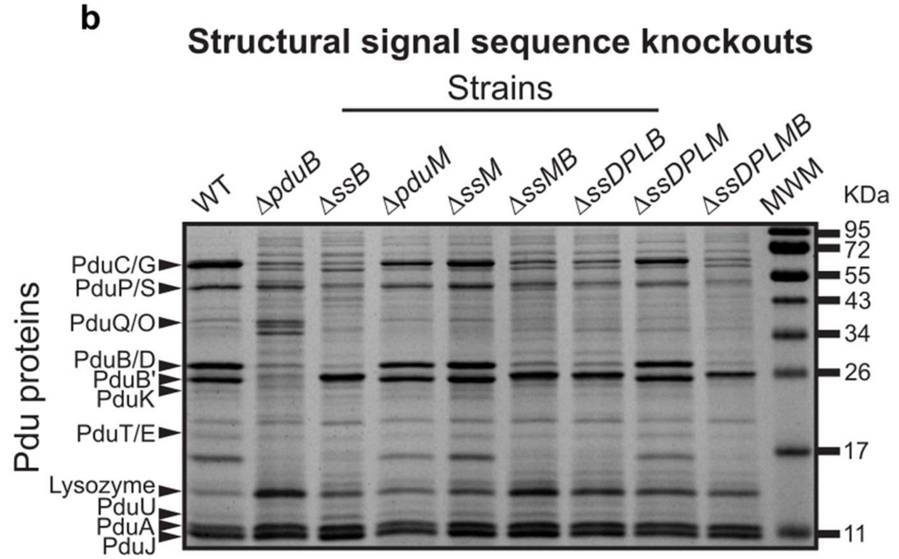

Supplementary Figure S9. Coomassie stained SDS-PAGE of MCPs purified from (a) enzymatic signal sequence knockout strains and (b) structural and structural + enzymatic signal sequence knockout strains. Bands corresponding to various Pdu proteins and lysozyme are labeled, and molecular weight markers (MWM) are included on the right side of the gels. Similar results were observed across three technical replicates.

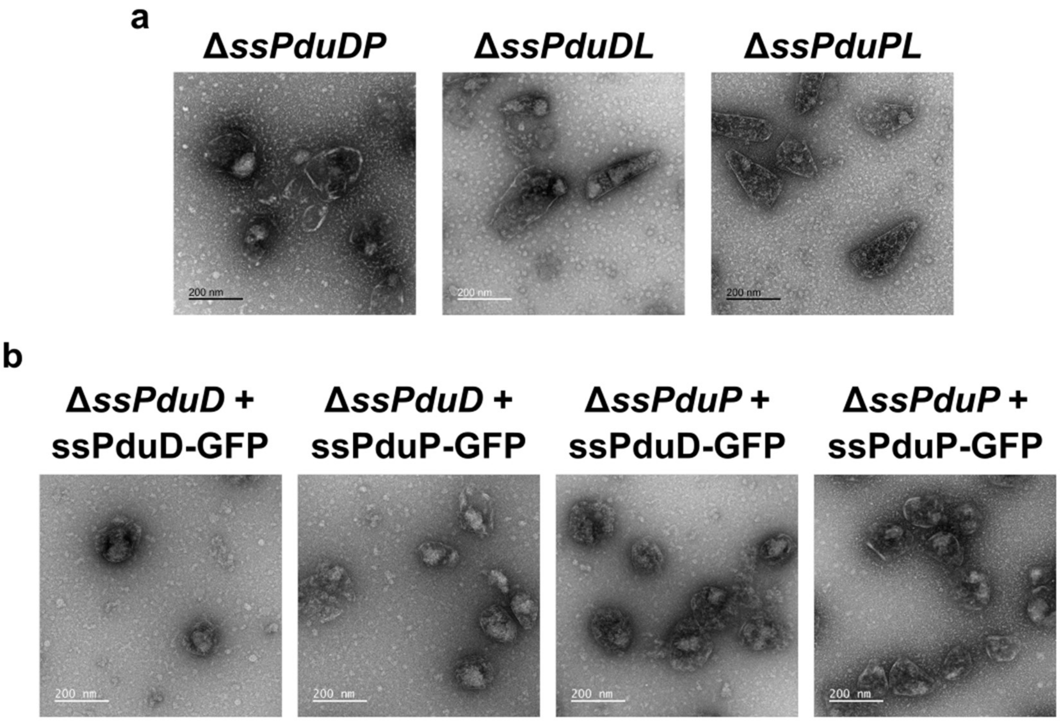

Supplementary Figure S10. Transmission electron micrographs of purified MCPs from (a) strains with two enzymatic signal sequences knocked out and (b) Δ*ssPduD* and Δ*ssPduP* complemented with overexpressed ssPduD-GFP and ssPduP-GFP. These images are representative of multiple images taken of the same sample, but due to time constraints, cost constraints, and a large number of samples, transmission electron micrographs were only taken of one biological replicate.

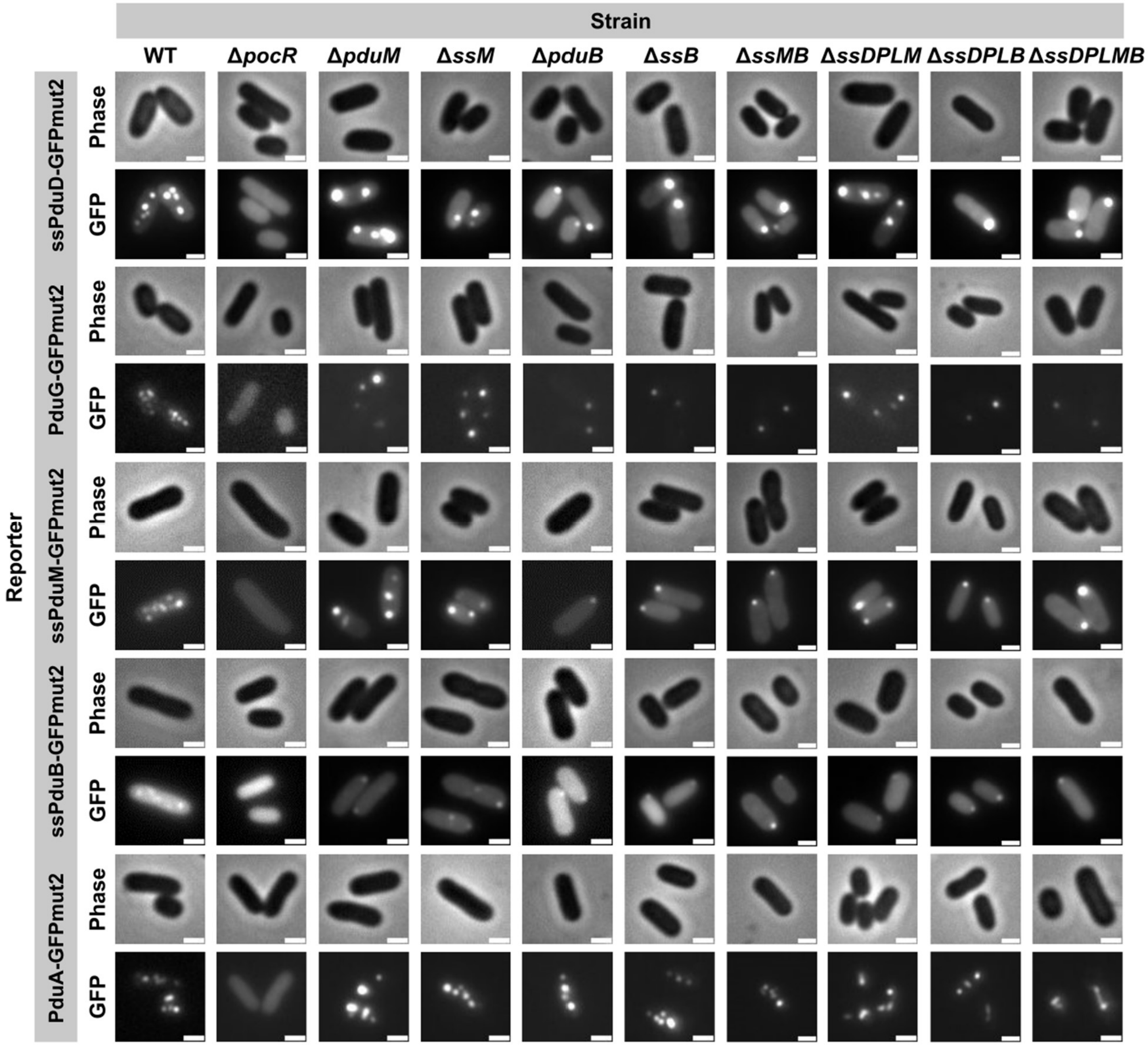

Supplementary Figure S11. Optical and fluorescence micrographs of Pdu MCP core and shell proteins and signal sequences fused to GFPmut2. These constructs were expressed in wild type (WT) *S. enterica*, in Δ*pocR*, an assembly-deficient strain in which all MCP formation is abolished, and in the structural and enzymatic + structural signal sequence knockout strains. All scale bars are 1 μm. Similar results were observed across at least three biological replicates of each strain.

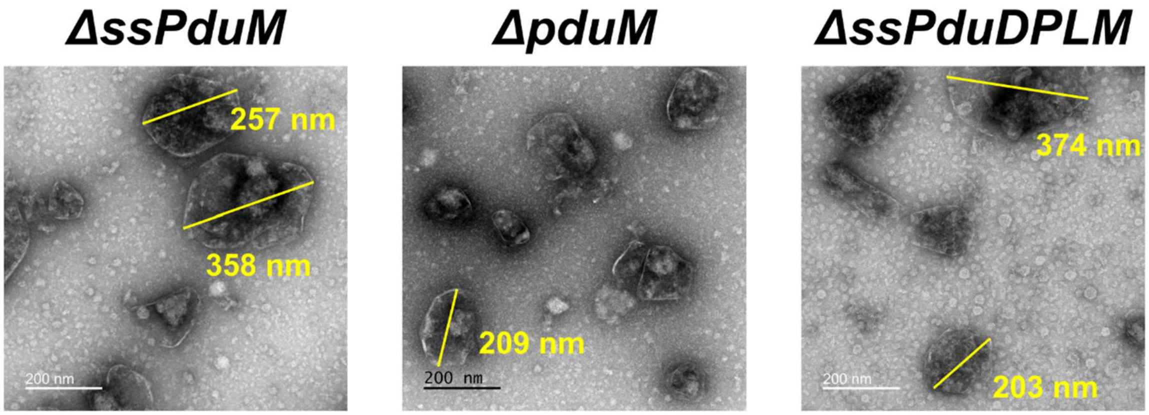

Supplementary Figure S12. Transmission electron micrographs of purified Δ*ssPduM*, Δ*pduM*, and Δ*ssPduDPLM* MCPs noting the sizes of MCPs over 200 nm in diameter. These images are representative of multiple images taken of the same sample, but due to time constraints, cost constraints, and a large number of samples, transmission electron micrographs were taken of only one biological replicate.

Supplementary References

1. Zimmermann L, Stephens A, Nam S-Z, Rau D, Kübler J, Lozajic M, Gabler F, Söding J, Lupas AN, Alva V. 2018. A completely reimplemented MPI bioinformatics toolkit with a new HHpred server at its core. J Mol Biol 430:2237–2243.

2. Jumper J, Evans R, Pritzel A, Green T, Figurnov M, Ronneberger O, Tunyasuvunakool K, Bates R, Žídek A, Potapenko A, Bridgland A, Meyer C, Kohl SAA, Ballard AJ, Cowie A, Romera-Paredes B, Nikolov S, Jain R, Adler J, Back T, Petersen S, Reiman D, Clancy E, Zielinski M, Steinegger M, Pacholska M, Berghammer T, Bodenstein S, Silver D, Vinyals O, Senior AW, Kavukcuoglu K, Kohli P, Hassabis D. 2021. Highly accurate protein structure prediction with AlphaFold. Nature 596:583–589.

3. Varadi M, Anyango S, Deshpande M, Nair S, Natassia C, Yordanova G, Yuan D, Stroe O, Wood G, Laydon A, Žídek A, Green T, Tunyasuvunakool K, Petersen S, Jumper J, Clancy E, Green R, Vora A, Lutfi M, Figurnov M, Cowie A, Hobbs N, Kohli P, Kleywegt G, Birney E, Hassabis D, Velankar S. 2022. AlphaFold Protein Structure Database: massively expanding the structural coverage of protein-sequence space with high-accuracy models. Nucleic Acids Res 50:D439–D444.

4. Pettersen EF, Goddard TD, Huang CC, Couch GS, Greenblatt DM, Meng EC, Ferrin TE. 2004. UCSF Chimera—A visualization system for exploratory research and analysis. J Comput Chem 25:1605–1612.
